# Immunization coverage and its determinant factors among children aged 12–23 months in Ethiopia: a systematic review, and Meta- analysis of cross-sectional studies

**DOI:** 10.1186/s12887-020-02163-0

**Published:** 2020-06-08

**Authors:** Akine Eshete, Sisay Shewasinad, Solomon Hailemeskel

**Affiliations:** 1 College of Health Sciences, Department of Public Health, Debre Berhan University, Debre Berhan, Ethiopia; 2 College of Health Sciences, Department of Nursing, Debre Berhan University, Debre Berhan, Ethiopia; 3 College of Health Sciences, Department of Midwifery, Debre Berhan University, Debre Berhan, Ethiopia

**Keywords:** Immunization-coverage, Vaccine, Children, Ethiopia, Systematic review, And meta-analysis

## Abstract

**Background:**

Immunization is the process by which a person is made immune or resistant to an infectious disease, typically by the administration of vaccine. Vaccination coverage for other single vaccines ranged from 49.1% for PCV to 69.2% for BCG vaccine. The vaccination coverage for basic vaccinations was 39.7% in Ethiopia. There have been epidemiological studies available on immunization in Ethiopia. Yet, these studies revealed a wide variation over time and across geographical areas. This systematic review and Meta-analysis aim to estimate the overall immunization coverage among 12–23 months children in Ethiopia.

**Methods:**

Cross-sectional studies that reported on immunization coverage from 2003 to August 2019 were systematically searched. Searches were conducted using PubMed, Google Scholar, Cochrane library, and gray literature. Information was extracted using a standardized form of Joanna Briggs Institute. The search was updated 20 Jan 2020 to decrease time-lag bias. The quality of studies assessed using Joanna Briggs Institute cross-sectional study quality assessment criteria. I-squared statistics applied to check the heterogeneity of studies. A funnel plot, Begg’s test, and Egger’s regression test was used to check for publication bias.

**Results:**

Out of 206 studies, 30 studies with 21,672 children with mothers were included in the Meta-analysis. The pooled full immunization coverage using the random-effect model in Ethiopia was 58.92% (95% CI: 51.26–66.58%). The trend of immunization coverage was improved from time to time, but there were great disparities among different regions. Amhara region had the highest pooled fully immunized coverage, 72.48 (95%CI: 62.81–82.16). The I^2^ statistics was I^2^ = 99.4% (*p* = 0.0001). A subgroup meta-analysis showed that region and study years were not the sources of heterogeneity.

**Conclusion:**

This review showed that full immunization coverage in Ethiopia was 58.92% (95% CI: 51.26–66.58%). The study suggests that the child routine immunization program needs to discuss this low immunization coverage and the current practice needs revision.

## Background

Immunization is the process by which a person is made immune or resistant to an infectious disease, typically by the administration of a vaccine. A vaccine is a non-pathogenic antigen that stimulates the body’s immune system to produce an antibody to protect the person against later infection. It is the most cost-effective public health intervention that can control and end life-threatening infectious disease [1, 2].

Vaccination has lowered the burden of infectious diseases since the start of the Expanded Program on Immunization (EPI) by the World Health Organization (WHO) in 1974, reducing mortality, morbidity, and saving resources [3–6]. WHO has estimated that 29% of under-five deaths could be prevented with existing vaccines, averting between 2 to 3 million deaths each year globally [7]. Worldwide immunization coverage showed improvement in the past years; however, the validity of the data for measuring change over time has been questioned [8]. Therefore, accurate immunization information is essential for decision-makers of the Expanded Program on Immunization (EPI) to track and improve performance [9].

The Expanded Programmed for Immunization (EPI) in Ethiopia, launched in 1980, has been one of the core priorities in the past Health Sector Development Programmes (HSDPs) and the current Health Sector Transformation Plan (HSTP). The country has mobilized women’s development armies or volunteers, health extension workers, and health facilities to deliver its immunization services. Improved district planning and management were started in 2011 to reach every district. Stationary, outreach, and mobile are the three important services delivery platforms for vaccination. The aim of launching this program was to increase the coverage of immunization by 10% annually. However, the coverage in the first 20 years was very low, although during the 1990s good progress was observed through Universal Child Immunization (UCI). Reaching every district approach has been implemented in Ethiopia, since, 2004 in districts with poor immunization coverage and high dropout rates. As a result, the coverage showed marked improvement, but there was a variation in coverage among regions. Now, reaching every district strategic approach is recast to reaching every child/community strategic approach to deal with inequities within districts [10].

Ethiopia’s national coverage of the third dose of the pentavalent, combined diphtheria, pertussis, tetanus, hepatitis B, and *Haemophilus influenza type B*, vaccine (Penta) at 12–23 months of age is 37%; and the dropout rate between the first and third doses of this vaccine was reported as 43% in 2013 [11].

The routine immunization coverage in Ethiopia has never reached the targeted figures and planned goals. Sustainable improvements in service delivery is needed to protect Ethiopian children from unnecessary suffering and deaths [12]. Similarly, according to the EDHS 2011 report, the coverage of EPI in Somali Region was low, and it showed that only 16.6% of them were fully immunized while 35.4% were unimmunized. These figures are two times lower than similar figures from other regions [13]. The main reasons behind this very low coverage where include a pastoral lifestyle and programmatic level to infrastructure conditions of the region, little commitment at all levels, lack of resource allocation, personnel and shortage of functional health facilities were also mentioned [2]. Complete immunization coverage was 38.5% at the national level and 45.8% in the Amhara region [14, 15].

In the search for effective ways to discuss low and stagnating vaccination rates and improve access to and utilization of immunization services, increased attention is being paid to the role of communities and community engagement strategy [16]. It is argued that communities should not be viewed as passive recipients of immunization services; rather, they need to be actively involved in shaping vaccination program [17].

### Objective and research question

The aim of this systemic review is synthesis and pooled level of full immunization coverage and its determinate factors among 12–23 months of children in Ethiopia. The research question is what is the level of full immunization coverage in Ethiopia?

## Methods

### Study settings

Ethiopia is one of the east African countries in the Horn of Africa. It covers an area of 1.104 million km^2^ and divided into 9 regions namely Tigray, Afar, Amhara, Oromia, Somali, Benishangul-Gumuz, Southern Nations Nationalities and People Region (SNNPR), Gambella, Harari, and two Administrative states (Addis Ababa city administration and Dire Dawa city administration).

### Criteria for considering studies for the review

#### Inclusion criteria

##### Selection of studies

Cross-sectional studies were extracted and two reviewers (SS, AE) employed the predetermined inclusion criteria to screen for relevant full-text cross-sectional studies. Both reviewers were blinded to journal, authors, and results. There were no conflicts between the two reviewers in last choice decisions. Studies were included for data extraction and analyses.

##### Inclusion criteria

Articles were included in this systematic review if they fulfilled the following criteria, study type: full-text cross-sectional articles written in English which have been published (since 2003) in peer-reviewed journals, primary journals, be on human subjects and 12–23 months age group.

##### Type of studies

All published cross-sectional studies including government reports related to the coverage of immunization status was included.

##### Study participants

Mothers/ caretakers with children aged 12–23 months, and in which immunization status was reported by card and mother recalled method.

##### Exclusion criteria

Citations without abstracts and/or full text, commentaries, anonymous reports, letters, duplicate studies were excluded.

### Search strategy and information sources

The database search had been structured using CoCoPop, where, Context (Ethiopia), condition (immunization coverage), Population (children aged 12–23 months). Notably, to fit the advanced PubMed database, the following search strategy applied: (Immunization OR Vaccination OR “Immunization Coverage” OR “Vaccination Coverage”) AND (Children OR “children aged 12-23 months”) AND (Determinant OR Determinants OR “Determinant factor” OR “Determinant factors” OR Factor OR Factors OR “Associated factor” OR “Associated factors”) AND (Ethiopia) AND full text [sb] AND (“2000/01/01”[PDat]: “2019/12/31”[PDat]) AND Humans [Mesh]. The presence of precursor systematic review and/or protocol on the topic of interest was checked on Cochrane database of a systematic review and Joanna Briggs Institute database of a systematic review. But, PROSPERO registration was not done.

An electronic database searches time was conducted using PubMed, Google Scholar, and Cochrane library and research gate from April 2019 to August 2019. To reduce time-lag bias, the search process was updated on 20 Jan 2020. The search focused on all published studies with epidemiological data of immunization coverage among children aged 12–23 months of children in Ethiopia. To find the relevant article, titles and abstracts of retrieved papers were exported to Endnote where duplicates were identified and removed by one investigator (SH). Full texts of peer-reviewed relevant articles were retrieved, assessed and their reference lists were hand-searched to show further relevant studies.

### Quality assessment tool

Retrieved studies were exported to endnote version 7 to remove duplicate studies. A search strategy was done by two of the investigators (SS and AE). Both the reviewers were blinded to journal, authors, and results. There were no conflicts between the two reviewers in final choice decisions. The selections of identified studies were done in two stages. In the first stage, a selection of relevant studies based on titles and abstracts. In the second stage, studies that met the inclusion criteria and the full paper found for detailed assessment based on the inclusion criteria were considered.

Two reviewers (SS and AE) performed the study eligibility assessment independently by using JBI checklists**.** A critical appraisal checklist for cross-sectional studies adopted by JBI and used to assess the overall methodological quality and evaluated the risk of bias (additional file 1). The methodological components assessed include: addressing the target population; data was extracted from the included cross-sectional studies: outcome measures counted magnitude of immunization coverage, and region, and publication year, Antenatal care, and institutional delivery. These data were then compiled into a standard table (Table 1). The two reviewers (SS, AE), who selected the proper studies also extracted the data and evaluated the risk of bias.

**Table 1:** Description of the included studies

Authors	Study area	Sample size	Measurement of outcome interest	Immunization Coverage of Children aged 12–23 Months	Other Main findings	JBI-Quality score
Animaw et al., 2014 [18]	SNNPR, Arba Minch town and Arba Minch Zuria district	630	Card plus mother recall method	461 (73.2%) were fully immunized	128 (20.3%) were partially and Vaccinated, and 41 (6.5%) were unvaccinated	88.9
Facha, 2015 [19]	SNNPR, Arba Minch Zuriya Woreda	210	Card plus mother recall method	112 (53.3%) were fully immunized	90 (42.9%) were partially vaccinated, and 8 (3.8%) did not take any vaccine	88.9
Meleko et al., 2017 [20]	SNNR, Mizan Aman town	322	Card plus mother recall method	136 (42.2%) were fully immunized	159 (49.4%) were partially vaccinated, and 27 (8.4%) were unvaccinated.	66.7
Tefera et al., 2018 [21]	SNNPR-Worabe, a town	484	Card plus mother recall method	297 (61.4%) were fully vaccinated.	187 (38.6%) were not fully immunized	66.7
Ayano, 2015 [22]	SNNPR,Hosanna Town	508	Card plus mother recall method	155 (30.51%) were fully vaccinated	325 (63.98%) were partially vaccinated, and 28 (5.51) were unvaccinated	66.7
Fite and Hailu, 2019 [23]	SNNPR Areka Town, Sothern Ethiopia	172	Not specified.	130 (75.6%) are fully vaccinated.	25 (14.5) were partially vaccinated, and 17 (10.1%) were unvaccinated	55.6
Hailu et al., 2019 [24]	SNNPR, Wonago district in southern Ethiopia	1116	Card plus mother recall method	585 (52.4%) were fully immunized	333 (29.8%) were partially immunized, and 158 (14.2%) were not immunized	100
Michael Mesfin, 2015 [25]	SNNPR, Yirgalem Town	473	Card plus mother recall method	367 (77.8) were fully immunized	96 (20) were partially immunized, and 10 (2.1%) were not immunized	77.8
Mohammed et al. 2013 [26]	Oromia, Kombolcha Woreda	694	Card plus mother recall method	159 (22.9%) completely immunized	-Of total 168 (24.2%) not immunized, and 367 (52.9%) partially immunized	66.7
Legesse and Dechasa, 2015 [27]	Oromia, Sinana district	591	Card plus mother recall method	- 454 (76.8%) were fully vaccinated	122 (20.6%) were partiallyVaccinated, and 15 (2.5%) were unvaccinated.	88.9
Melese Girmaye, et al. 2019 [28]	Oromia, Wayu-Tuka District	436	Card plus mother recall method	Fully vaccination coverage was 73.9%	109 (25%) were partially vaccinated, and (1.1%) were unvaccinated.	66.7
Sheka Shimelis, 2018 [29]	Oromia, in Serbo Town	260	Card plus mother recall method	119 (48.8%) were fully immunized	−45.5% (126) were incompletely vaccinated and 5.7% (14) did not take any vaccination.	88.9
Udessa, 2018 [30]	Oromia, Wadera District	440	Card plus recall method	184 (41.4%) were fully vaccinated	Among the total, 26 (5.9%) of the children were unvaccinated.	66.7
Etana and Deressa, 2012 [31]	Oromia, Ambo Woreda	536	Card plus mother recall method	191 (35.6%) were fully vaccinated	218 (40.6) were partially vaccinated, and 127 (23.7%) were unvaccinated.	88.9
Wado et al., 2014 [32]	Oromia southwestern Ethiopia	889	Immunization coverage by card	329 (37%) were fully vaccinated	361 (40.6%) were partially vaccinated, and 199 (22.4%) were unvaccinated.	77.8
Toyeb Yasine, 2015 [33]	Oromia, Tehulederie district	639	Immunization coverage by card	531 (83.1) were fully vaccinated	94 (14.7%) were partially vaccinated, and 14 (2.2%) were unvaccinated.	77.8
Kassahun et al., 2015 [34]	Amhar, Lay Armachiho District	751	Card plus mother recall method	571 (76.03%) were fully vaccinated	21.67 were partially vaccinated, and (2.3) not vaccinated at all	88.9
Gualu and Dilie, 2017 [35]	Amhara, Debre Markos Town	288	Not specified	264 (91.7%) were fully vaccinated	19 (6.6%) were partially vaccinated, and 5 (1.7%) were not vaccinated at all.	55.6
Mastewal Worku Lake et al., 2016 [36]	Amhara, Dessie Town,South Wollo Zone	724	Card plus mother recall method	472 (65.2%) were fully vaccinated	130 (17.9%) were partially vaccinated, and 252 (34.8%) never get vaccine	77.8
Ayal D, 2014 [37]	Amhara, Mecha district	497	Card plus mother recall method	245 (49.3%) were fully vaccinated	244 (49.1%) were partially vaccinated, and 8 (1.6%) have never been vaccinated.	66.7
Abebe et al., 2019 [38]	Amhara, Woldia Town	389	Card plus mother recall method	343 (87.7%) children were fully immunized	46 (11.8%) were partially vaccinated	66.7
Tadesse daget, 2018 [39]	Amhara, Bahirdar town	846	Card plus mother recall method	494 (58.4%) were fully vaccinated	144 (17%)were partially vaccinated and 208 (24.6%) were not vaccinated at all	88.9
Mekonnen et al., 2019 [40]	Minjar-Shenkora district	566	mother’s/caregivers’ report	428 (75.6%) were fully vaccinated	105 (18.5%) were partially vaccinated, and 33 (5.9%) were not vaccinated at all	88.9
Ayenew Engida, 2019 [41]	Amhara, Gondar city administration	301	Card plus mother recall method	228 (75.7) were fully vaccinated	73 (24.3%) were partially vaccinated	77.8
Girmay and Dadi, 2019 [42]	Tigria, Sekota Zuria district.	620	Card plus mother recall method	480 (77.4%) of them were fully immunized	15.5% (96/620) were partially immunized, and 44 (7.1%) did not received vaccin	77.8
Teklay Kidane, 2004 [43]	Tigria regional State	110	Card plus mother recall method	83 (75.5%) were fully vaccinated	27 (24.5%) were partially immunized	77.8
Mohamud et al., 2014 [44]	Somali National Regional State	582	Card plus mother recall method	213 (36.6%) were fully vaccinated	221 (37.9) were partially vaccinated, and 148 (25.4%) not vaccinated at all	88.9
Yihunie Lakew, 2015 [45]	National survey	1927	Card plus mother recall method	468 (24.3%) were fully vaccinated	1170 (60.7%) were partially vaccinated, and 289 (15%) were not vaccinated at all	77.8
Koku Sisay, 2019 [46]	National survey	1909	Card plus mother recall method	38.3% are fully vaccinated	61.7% were partially vaccinated.	88.9
Abebech Asmamaw, 2016 [47]	National survey	4983	Card plus mother recall method	1296 (26%) are fully vaccinated	–	88.9

### Data extraction

A standardized data extraction form of JBI was used to extract the necessary data. The data extraction tool was piloted by considering the inclusion criteria to check consistency and to make sure that all the relevant information was captured. The extraction tool includes the title of the study, the first author’s name, and year of publication, study area (region) and all other important information. During the extraction process, data discrepancy among data extractors was resolved by referring back to the original study.

**Fig. 1:**
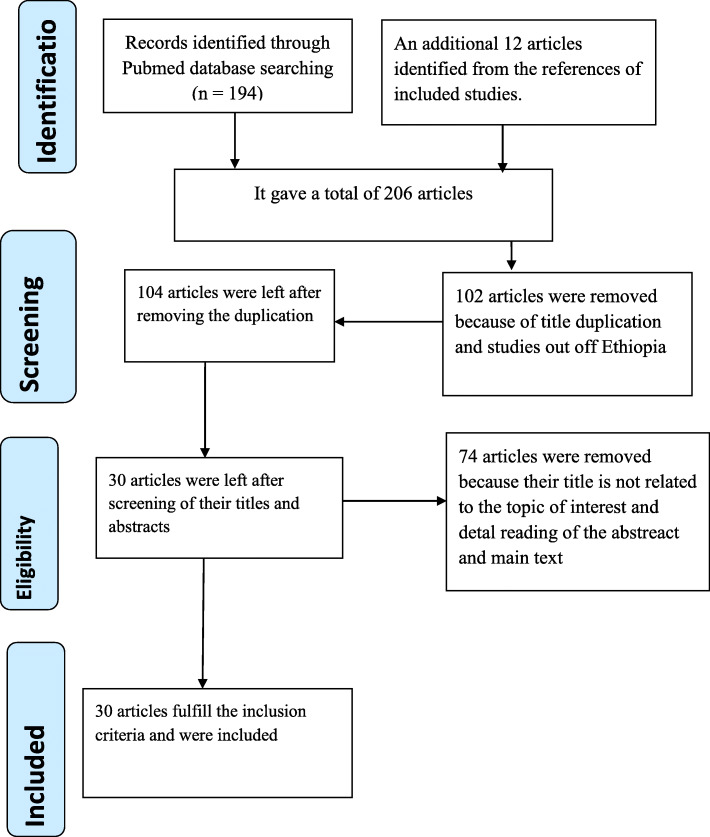
The PRISMA flow diagram of identification and selection of studies for the systematic review and meta-analysis

The third reviewer (SH) negotiated any discrepancy between the two authors. In other words, the papers were given to the third reviewer for consensus while a discrepancy in the decision process. The screening and selection process of the reviewed articles was summarized using the PRISMA flow chart (Fig. 1 [48]).

### Outcome measures (fully immunizations/ immunization coverage)

Fully immunization coverage was the primary interest of this review, which was measured if the child took all recommended vaccines according to the Ethiopian EPI schedule. According to the WHO guideline “complete or full immunization” coverage is defined as a child has received a *BCG vaccine*, three doses of penta vaccine such as *diphtheria, pertussis, tetanus, hepatitis B* and *Haemophilus influenza type B*; at least three doses of polio vaccine, 3 doses of PCV vaccine, 2 doses of Rota vaccine and one dose of measles vaccine. It was assessed by vaccination card plus mothers recall [49].

### Data synthesis and statistical analysis

Data was analyzed using the ‘meta’ packages of the Stata software (version 11.0). Unadjusted prevalence was recalculated based on crude numerators and denominators provided by each study and joined to calculate the pooled estimates. The quantitative data synthesis method was used to present extracted data from each study. Heterogeneity among the studies was evaluated using the χ2 test on Cochrane’s Q statistic [50], and I-square estimate greater than 75% was considered as indicative of moderate to high levels of heterogeneity [51]. Subgroup analysis was done to explore differences in outcomes according to a study area, study region, publication year. The funnel plot and Egger’s test were used to check the presence of publication bias [52]. A *p*-value < 0.05 on the Egger test was considered indicative of publication bias.

## Results

### Description of the included studies

The search strategy retrieved 206 studies from PubMed, Cochrane library, Google Scholar and gray literature. About 102 articles were excluded because of duplication matters and studies out of Ethiopia. After removing duplicates, a total of 74 articles were removed by reading title and abstract of the studies. Finally, 30 studies were screened for full-text review and used for quantitative analysis (Fig. 1).

### Characteristics of included studies

Full-text cross-sectional articles written in English and published from 2003 to 2019 years were studied in a different part of Ethiopia. Of 30 studies, eight of them were done in Amhara region, eight in the Southern Nation Nationality People Region (SNNPR), eight in Oromia region, two in Tigray, three studies at national level study, and one in Somali National Regional State. In the included studies, the sample sizes were ranges from 172 to 3762. A total of 21,562 children aged 12–23 months were included in all studies. A summary of all relevant features and main findings of the including studies were presented in (Table 1).

### Fully immunization coverage among children 12–23 months in Ethiopia

In the included studies, full immunization coverage ranges from 22.9% [26] to 91.7% [35]. Among the total reviewed studies, in fifteenth studies, full immunization coverage was dominantly reported within the ranges of 22.9 to 58.4%. In 12, included individual studies, most children were fully immunized that reported within the range from 61.4 to 77.8%. In three, included studies, full immunization coverage was high which accounts for 87.7 to 91.7% (Table 1).

### Partial-immunization coverage among children 12–23 months in Ethiopia

Partial immunizations were reported by 26 studies. The magnitude of partial immunization ranges from 63.98% at SNNPR, hosanna town to 6.6% at Amhara region, Debre Markos Town (Table 1).

### Non-immunization coverage among children 12–23 months in Ethiopia

No immunizations were reported by 24 studies. The magnitude of never immunized children was range from 34.8% at Amhara Region, Dessie Town to (1.1%) at Oromia region, Wayu-Tuka District (Table 1).

### Meta-analysis results

The drive of this meta-analysis was to estimate the pooled level fully immunization coverage among children 12–23 months in Ethiopia, by using proportions. A total of 30 studies met the inclusion criteria for meta-analysis.

### Fully immunization coverage among children 12–23 months in Ethiopia

**Fig. 2:**
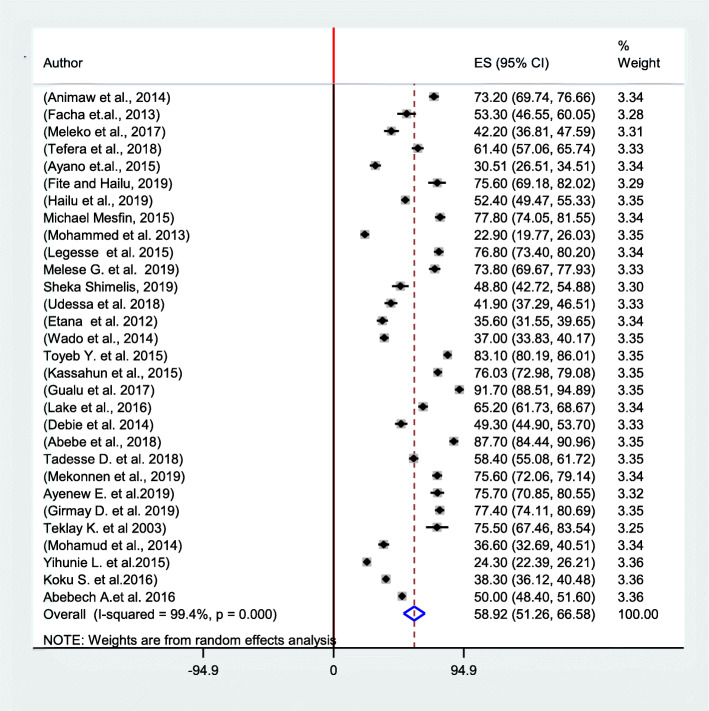
Proportion of fully immunization coverage among children 12–23 months in Ethiopia from 2003 to 2019

A total of 30 studies were included in this meta-analysis. The estimated overall pooled proportion of fully immunized children in Ethiopia were 58.92, (95%CI: 51.26–66.58) (Fig. 2).

**Fig. 3:**
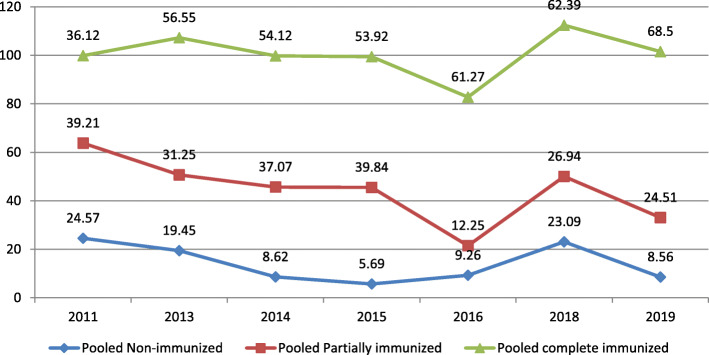
Trend of immunization coverage among children 12–23 months in Ethiopia from 2003 to 2019

In the regional subgroup analysis, Amhara region had the highest proportion of fully immunized children at 72.48(95%CI: 62.81–82.16), followed by SNNPR 58.30(46.42–70.18) and Oromia region 52.50 (95%CI; 35.08–69.91). The highest proportion of pooled fully immunization coverage was observed in the year 2019, 68.50, (95% CI: 59.17–77.83), but almost similar in the year 2016, 61.27, (95%CL: 41.43–81.08) and 2018, 62.39, (95% CL: 43.38–81.39) (Table 2 and Fig. 3).

**Table 2:** Immunization coverage in Ethiopia among children age 12–23 months in Ethiopia from 2003 to 2019

Variables	Coverage% (95% CI)	Heterogeneity	No. studies
**Immunization coverage**			
Full immunization	58.92 (51.26–66.58)	I2 = 99.4%, p = 0.000	30
Partial immunization	30.80 (23.91–37.65)	I2 = 99.1%, *p* = 0.000	25
Non-immunization	12.87 (9.77–15.96)	I2 = 98.5%, *p* = 0.000	24
**Regional status**			
Oromia region	52.50 (35.08–69.91)	I2 = 99.4%, p = 0.000	8
Amhara region	72.48 (62.81–82.16)	I2 = 98.3%, p = 0.000	8
SNNPR	58.30 (46.42–70.18)	I2 = 98.4%, p = 0.000	8
National level study	37.54 (21.99–53.09)	I2 = 99.5%, p = 0.000	3
**Complete/full / Immunization**			
2011	36.12 (33.30–38.93)	I2 = 0.001%, *p* = 0.728	2
2013	56.55 (28.16–84.94)	I2 = 99.6%, p = 0.000	4
2014	54.12 (29.03–79.21)	I2 = 99.4%, p = 0.000	3
2015	53.92 (21.19–86.66)	I2 = 99.8%, p = 0.000	4
2016	61.27 (41.43–81.08)	I2 = 99.6%, p = 0.000	4
2018	62.39 (43.38–81.39)	I2 = 99.0%, p = 0.000	4
2019	68.50 (59.17–77.83)	I2 = 97.3%, p = 0.000	7
**Partially immunization**			
2011	39.21 (36.36–42.07)	I2 = 0.001%, *p* = 0.343	2
2013	31.25 (11.17–51.33)	I2 = 99.1%, p = 0.000	3
2014	37.07 (21.04–53.09)	I2 = 98.4%, p = 0.000	3
2015	39.84 (13.49–66.19)	I2 = 99.7%, p = 0.000	4
2016	12.25 (1.18–23.33)	I2 = 96.7%, p = 0.000	2
2018	26.94 (6.98–46.9)	I2 = 99.1%, p = 0.000	3
2019	24.51 (16.96–32.09)	I2 = 95.9%, p = 0.000	6
**Non-immunization**			
2011	24.57 (22.04–27.09)	I2 = 0.001%, *p* = 0.509	2
2013	19.45 (7.80–31.10)	I2 = 99.1%, p = 0.000	3
2014	8.62 (0.57–16.67)	I2 = 99.0%, p = 0.000	3
2015	5.69 (0.09–11.29)	I2 = 98.5%, p = 0.000	4
2016	9.26 (5.64–24.15)	I2 = 98.9%, p = 0.000	2
2018	23.09 (4.77–41.42)	I2 = 95.9%, p = 0.000	3
2019	8.56 (5.02–12.11)	I2 = 90.6%, p = 0.000	6

### Partial and non-immunization coverage among children 12–23 months in Ethiopia

The pooled proportion of partially immunized children was 31.05% (95% CI: 24.00–38.10). The highest pooled proportion of partial immunization coverage was observed in the year 2015, 39.84 (95%CI; 13.49–66.19), but lower coverage was observed in the year 2019, 24.51, (95%CI; 16.96–32.09) (Table 1). The pooled proportion of non-immunization of children was 12.87(95%CI; 9.77–15.96) (Table 2 and Fig. 3).

### Factors associated with fully immunization coverage among children age 12–23 months

In this meta-analysis, urban residence OR:1.75; (95% CI: 1.42–2.17), maternal education OR:2.29;(95% CI:1.19–2.75), ANC follows ups OR: 2.38;(95% CI:2.06–2.76), delivery at health facilities OR:1.87;(95%CI:1.68–2.09), maternal TT vaccination OR:1.40;(95%CI:1.21–1.64), PNC follows OR:1.44;(95%CI:1.14–1.82), knowledge about immunization OR: 3.83;(95%CI: 2.88–5.10), mother knowing the schedule of vaccination OR:2.06;(95%CI:1.56–2.71), attitude towards immunization OR:1.86;(95%CI:1,04–5.33), mother who visited by HEW OR:2.23; (95%CI:1.63–3.04) were significantly associated with full immunization (Table 3).

**Table 3:** factors associated with fully immunization coverage among children age 12–23 months in Ethiopia from 2003 to 2019

Variables	OR, 95% CI	Heterogeneity	Number of studies
Educated mothers	2.29 (1.19–2.75)	I^2^ = 68.4, *p* = 0.004	7
Knowledgeable about immunization	3.83 (2.88–5.10)	I^2^ = 64.1, *p* = 0.025	5
Mother who had ANC visit	2.38 (2.06–2.76)	I^2^ = 71.0, p = 0.0001	10
Favorable attitude towards immunization	1.86 (1,04–5.33)	I^2^ = 0.0, *p* = 0.445	3
Mother who delivered at health institution	1.87 (1.68–2.09)	I^2^ = 57.4, *p* = 0.002	17
Mother who visited by HEW	2.23 (1.63–3.04)	I^2^ = 0.0, *p* = 0.592	3
Mother who lived at urban kebeles	1.75 (1.42–2.17)	I^2^ = 0.0, *p* = 0.580	5
Mother who taken TT vaccination	1.40 (1.21–1.64)	I^2^ = 47.8, *p* = 0.105	5
Mother who had PNC visit	1.44 (1.14–1.82)	I^2^ = 46.1, *p* = 0.116	5
Mother knowing the schedule of vaccination	2.06 (1.56–2.71)	I^2^ = 0.0, *p* = 0.523	3

### Evaluation for publication bias

The presence of heterogeneity among the studies was tested using I-squared statistics. I-squared (I^2^) statistics for full immunization coverage was (I^2^ = 99.4%) (*p* = < 0.0001), which indicates as there is high heterogeneity between studies. A *p*-value of < 0.0001, indicates the presence of significant heterogeneity among the included studies. The weights of the studies were reported from the random-effect model which ranged from 3.42 to 3.45% (Fig. 1).

We further conducted a subgroup meta-analysis to identify the source of this high heterogeneity using region and publication year. The I^2^ value for the region subgroup test was found to be 99.5% (*p*-value < 0.0001) which indicated the presence of heterogeneity between studies (Table 2).

**Fig. 4:**
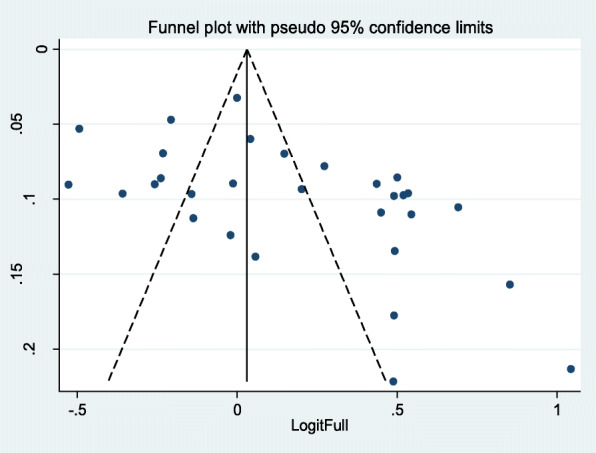
Funnel plot of effect estimates against standard error of log estimate

The funnel plot is to be unsymmetrical and the distribution of studies indicates for the presence of heterogeneity. More studies are found on both sides of the funnel plot margin (Fig. 4). Egger’s test was performed, and the test showed there was a significant bias among studies (overall test: intercept = 3.92, 95% CI; 12.32–39.37and *p*-value = 0.001).

**Fig. 5:**
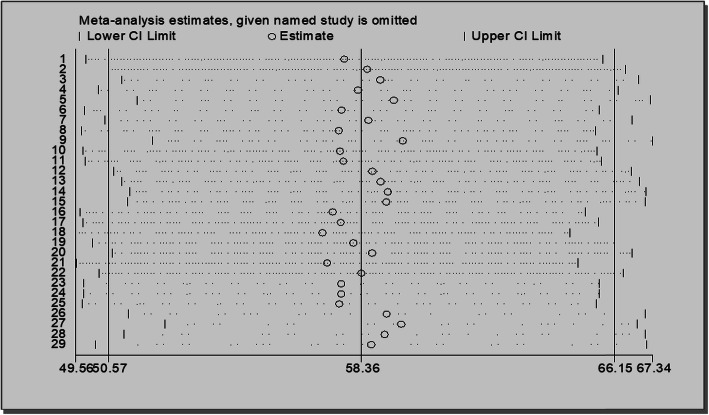
Plot of sensitivity analysis to assessing the influence of individual study

Sensitivity analysis has been performed to find the influence of each study on the estimates. The plot provides the omitted study on both sides of the margin that indicates there were studies that affect the estimates (Fig. 5).

## Discussion

Immunization has been one of the most cost-effective health interventions worldwide, through which several serious childhood diseases have been successfully prevented or eliminated. However, vaccination could only become more effective if the child is given a chance to receive the full course of recommended vaccination doses [53].

In this meta-analysis, the proportion of pooled full immunization coverage among children in Ethiopia using the random-effect model was 58.92% (95%CI: 51.26–66.58%). The five consecutive Ethiopia Demographic health survey studies, immunization coverage’s were 14% in 2000, 20% in 2005, 24% in 2011, 39% in 2016 and 43% in 2019 [53, 54]. However, this pooled full immunization coverage indicates less promising to meet the 2020 health sector transformation plan of reaching immunization coverage to 95% in Ethiopia [55].

Understanding the barriers of immunization coverage was critical to formulating effective policies and programs. Lessons from different studies in Ethiopia revealed that fear of immunization side effects, lack of awareness about vaccination, take part negative attitude for the benefit of vaccination, child was sick, unavailability of vaccine, place of immunization too far, due to family health problem, absence of vaccinator, inconvenience vaccination schedule, far distance from health facility, wrong ideas about contraindications and religious, and custom restriction, were major causes for never vaccinated. Therefore, immunization programs should go beyond offering vaccination at health sectors [5] and strengthening collaboration to meet the coverage of all recommended basic vaccines in Ethiopia. Besides, reaching every community strategy (door to door immunization strategy) is an innovative approach that seeks to improve immunization coverage at health facilities [56]. The key goal of the immunization agenda by 2030 is to make vaccination available to everyone and everywhere [57].

This current proportion of pooled full immunization coverage was 58.92% (95%CI: 51.26–66.58%), other systematic review and meta-analysis in Nigeria showed that full immunization coverage was (34.4%) [58], and a national study conducted in Myanmar was (55.4%) [59], national health survey in Malaysia was (86.4%) [60]. In identified studies, forgetting the appointment date, lack of awareness about vaccination, absence of health worker on health facility, place and/or time of vaccination unknown, postponed until another time, fear of immunization side effect, mother too busy, long waiting time, child sick in the time of vaccination, far distance of immunization site, unaware of when to return 2nd or 3rd dose, don’t know next schedule and place, the experience of child sickness with earlier vaccination, disrespectful behavior of health professionals were major causes for incomplete immunization. Thus, the findings highlight that immunization coverage is not an exceptional problem of Ethiopia, it also a problem of other countries that demanding a strong immunization program.

To achieve complete immunization coverage across all regions in Ethiopia, policymakers should design different interventions. For example, the success of immunization services is closely linked to the perceived quality of health services by the public. Health workers engaged in vaccination needed to be skilled in all aspects of vaccine administration, cold chain, and logistics. Regular training and supervision should emphasize these areas [5].

Understanding the determinants of immunization coverage is vital for the improvement of immunization status and identifies area that need to be focused by health care providers and policy-makers. In this meta-analysis, urban residence, maternal education, ANC follows ups, delivery at health facilities, maternal TT vaccination, PNC follows, knowledge about immunization, mother knowing the schedule of vaccination, attitude towards immunization, Mother who visited by HEW were significantly associated with full immunization.

## Conclusions

The pooled proportion of immunization coverage in Ethiopia was 58.92% (95%CI: 51.26–66.58%). It was lower compared with 2020 governmental plan of immunization coverage to be 95%, but the proportion of pooled fully immunization coverage was improved from time to time. In this review, there were great disparities in immunization coverage among different regions in Ethiopia.

### Implications for practice

Even though improving childhood vaccine coverage is a major priority health agenda in Ethiopia, immunization coverage remains a significant health problem [55]. In this review, the finding indicates that immunization coverage was improved from time to time, but the proportion of full immunization status still lower. In light of these challenges, the country needs to strengthen the implementation of the health extension program, implementation of reaching every district approach, strengthen the health development army in the community, and the government needs to work with the private sector and nongovernmental providers that will improve vaccination coverage in the country. Strategies are needed to make sure that private and public providers implement to reduce barriers and missed opportunities for vaccination [61].

The government needs to build capacity in their communities that emphasize the benefits of full immunization for their children. Individuals and communities should understand the benefits and participate in the decision-making, and delivery process. The community leaders should promote and collaborate closely with local health staff in outreach activities in the communities. However, the growing complexity of immunization programs increases the need for a well-trained, capable health workforce [62]. Children who received other health interventions were more likely to be fully immunized [63]. Therefore, immunization services should integrate with maternal health services in the actual service delivery setups that make it convenient for patients (mothers and their children) to receive vaccinations at primary healthcare settings in Ethiopia.

Lastly, understanding the determinants of immunization coverage is vital for the improvement of immunization status. And also the finding suggests that improved health education and service expansion to remote areas, strength the local specific health service and creating awareness of mothers to complete recommended doses of vaccination are necessary to step immunization access.

## Supplementary information

**Additional file 1.**

## Data Availability

The authors confirm that all relevant data was included in the manuscript.

## Abbreviations

DPT: Diphtheria, Pertussis, and Tetanus
EDHS: Ethiopia Demographic and Health Survey
EPI: Expanded Program on Immunization
HSTP: Health Sector Transformation Plan
HTA: Health Technology Assessment
CINAHL: Cumulative Index to Nursing and Allied Health Literature
AMED: Allied and Complementary Medicine
WHO: World Health Organization
MeSH: Medical Subject Heading
JBI-DSRIR: Joanna Briggs Institute Database of a Systematic Review and Implementation Reports
PRISMA: Preferred Reporting Items for Systematic Review and Meta-Analysis
