# Supplementary material for: Immunization coverage and its determinant factors among children aged 12–23 months in Ethiopia: a systematic review, and Meta- analysis of cross-sectional studies
Source: BMC Pediatr. 2020 Jun 8;20:283. doi: 10.1186/s12887-020-02163-0 (PMC7278125; doi:10.1186/s12887-020-02163-0)
Supplement: Supplementary file 1 — Additional file 1. [file 12887_2020_2163_MOESM1_ESM.docx]

Additional file 1: Risk of bias within studies using the Joanna Briggs Institute criteria’s

| Included Studies | JBI quality assessment criteria’s | | | | | | | | | Total Score % |
| --- | --- | --- | --- | --- | --- | --- | --- | --- | --- | --- |
|  | Q1 | Q2 | Q3 | Q4 | Q5 | Q6 | Q7 | Q8 | Q9 |  |
| Animaw et al., 2014 | Y | Y | Y | Y | Y | Y | N | Y | Y | 88.9 |
| Facha , 2013 | Y | Y | N | Y | Y | Y | Y | Y | Y | 88.9 |
| Meleko et al., 2017 | Y | Y | N | N | Y | Y | Y | N | Y | 66.7 |
| Tefera et al., 2018 | Y | N | N | N | Y | Y | Y | Y | Y | 66.7 |
| Ayano, 2015 | Y | Y | Y | N | Y | Y | U | N | Y | 66.7 |
| Fite and Hailu, 2019 | Y | Y | N | N | Y | U | Y | N | Y | 55.6 |
| Hailu et al., 2019 | Y | Y | Y | Y | Y | Y | Y | Y | Y | 100 |
| Michael Mesfin, 2015 | Y | U | N | Y | Y | Y | Y | Y | Y | 77.8 |
| Mohammed et al 2013 | Y | Y | Y | U | Y | N | Y | U | Y | 66.7 |
| Legesse and Dechasa, 2015 | Y | Y | Y | Y | Y | Y | N | Y | Y | 88.9 |
| Melese Girmaye Negero., et al.2019 | Y | U | N | Y | Y | Y | U | Y | Y | 66.7 |
| Sheka Shimelis, 2019 | Y | Y | N | Y | Y | Y | Y | Y | Y | 88.9 |
| Udessa, 2018 | Y | U | N | Y | Y | U | Y | Y | Y | 66.7 |
| Etana and Deressa, 2012 | Y | Y | Y | Y | Y | Y | U | Y | Y | 88.9 |
| Wado et al., 2014 | Y | Y | Y | Y | Y | U | U | Y | Y | 77.8 |
| Toyeb Yasine, 2015 | Y | Y | Y | U | Y | Y | N | Y | Y | 77.8 |
| Kassahun et al., 2015 | Y | Y | Y | Y | Y | Y | U | Y | Y | 88.9 |
| Gualu and Dilie, 2017 | Y | U | N | Y | Y | U | U | Y | Y | 55.6 |
| Lake et al., 2016 | Y | Y | Y | U | Y | Y | N | Y | Y | 77.8 |
| Debie and Taye, 2014 | Y | U | N | Y | Y | Y | U | Y | Y | 66.7 |
| Abebe et al., 2018 | Y | Y | N | Y | Y | U | U | Y | Y | 66.7 |
| Tadesse daget, 2018 | Y | Y | Y | Y | Y | U | Y | Y | Y | 88.9 |
| Mekonnen et al., 2019 | Y | Y | Y | Y | Y | Y | U | Y | Y | 88.9 |
| Ayenew Engida, 2019 | Y | Y | N | Y | Y | U | Y | Y | Y | 77.8 |
| Girmay and Dadi, 2019 | Y | Y | Y | U | Y | Y | U | Y | Y | 77.8 |
| Teklay Kidane, 2003 | Y | Y | Y | Y | Y | U | U | Y | Y | 77.8 |
| Mohamud et al., 2014 | Y | Y | Y | Y | Y | Y | U | Y | Y | 88.9 |
| Yihunie Lakew, 2015 | Y | Y | Y | U | Y | U | Y | Y | Y | 77.8 |
| Koku Sisay, 2016 | Y | Y | Y | U | Y | Y | Y | Y | Y | 88.9 |
| Abebech Asmamaw, 2016 | Y | Y | Y | Y | Y | U | Y | Y | Y | 88.9 |
| Note: Y - Yes, N - No, U – Unclear, NA-Not applicable  Q1= Was the sample frame appropriate to address the target population?  Q2= Were study participants sampled in an appropriate way?  Q3= Was the sample size adequate?  Q4= Were the study subjects and the setting described in detail?  Q5= Was the data analysis conducted with sufficient coverage of the identified sample?  Q6= Were valid methods used for the identification of the condition?  Q7= Was the condition measured in a standard, reliable way for all participants?  Q8= Was there appropriate statistical analysis?  Q9= Was the response rate adequate, and if not, was the low response rate managed appropriately? | | | | | | | | | | |
